# Trophoblast Exosomal UCA1 Induces Endothelial Injury through the PFN1-RhoA/ROCK Pathway in Preeclampsia: A Human-Specific Adaptive Pathogenic Mechanism

**DOI:** 10.1155/2022/2198923

**Published:** 2022-09-15

**Authors:** Suwen Wu, Yutong Cui, Huanqiang Zhao, Xirong Xiao, Lili Gong, Huangfang Xu, Qiongjie Zhou, Duan Ma, Xiaotian Li

**Affiliations:** ^1^Obstetrics and Gynecology Hospital of Fudan University, Shanghai, China; ^2^Key Laboratory of Metabolism and Molecular Medicine, Department of Biochemistry and Molecular Biology, School of Basic Medical Sciences, Fudan University, Shanghai, China; ^3^Institutes of Biochemical Sciences, Fudan University, Shanghai, China

## Abstract

Preeclampsia is regarded as an evolution-related disease that has only been observed in humans and our closest relatives, and the important factor contributing to its pathogenesis is endothelial dysregulation secondary to a stressed placenta. Hypoxia-inducible factor 1 subunit alpha (HIF1*α*), a highly conserved molecule in virtually all mammals, is regarded as a crucial regulator of the hypoxia adaptation and evolution. Persistent high expression of HIF1*α* in the placenta is one of the pathogenic mechanisms of preeclampsia. Therefore, human-specific molecules should link increased HIF1*α* to preeclampsia. We reported that urothelial cancer associated 1 (UCA1) is a potential mediator because it is a human-specific long noncoding RNA (lncRNA) that is upregulated in placental tissues and maternal serum from women with preeclampsia and is regulated by HIF1*α*. The cellular HIF1*α*-UCA1 pathway promoted the adaptation of trophoblasts to hypoxia by inducing vascular endothelial growth factor (VEGF) secretion and changes in the levels of key enzymes in glycolysis. On the other hand, circulating exosomal UCA1 secreted from stressed trophoblasts induced vascular endothelial dysfunction, especially excess ROS production, as measured by exosome extraction and a coculture system. At the molecular level, UCA1 physically bound to ubiquitin-specific peptidase 14 (USP14), which is a deubiquitinating enzyme, and UCA1 functioned as a scaffold to recruit USP14 to profilin 1 (PFN1), an actin-binding protein contributing to endothelial abnormalities and vascular diseases. This ternary complex inhibited the ubiquitination-dependent degradation of PFN1 and prolonged its half-life, further activating the RhoA/Rho-kinase (ROCK) pathway to induce ROS production in endothelial cells. Taken together, these observations suggest a role for the evolution-related UCA1 in the HIF1*α*-induced adaptive pathogenic mechanism of preeclampsia, promoting the survival of hypoxic trophoblasts and injuring maternal endothelial cells.

## 1. Introduction

Preeclampsia is an enigmatic and evolution-related disease that occurs in humans and our closest relatives, such as gorillas and chimpanzees [1, 2], and affects an estimated 4-5% of pregnancies worldwide [3–5]. Impaired trophoblast invasion and failed spiral artery remodeling in early gestation lead to reduced uteroplacental blood flow and a stressed placenta; the latter triggers the dysfunction of maternal peripheral endothelial cells, causing a systemic inflammatory response and clinical syndromes centered on maternal hypertension, but the molecular mechanism of this second stage is still not well understood. Given the species specificity of this disease, focusing on evolution-related molecules may be an interesting entry point.

In mammalian evolution, hypoxia-inducible factor 1 subunit alpha (HIF1*α*) is a crucial regulator of cellular adaptations [6, 7], including glucose metabolism, cell proliferation, erythropoiesis, and vascularization. HIF1*α* also regulates the biofunctions of trophoblasts [8, 9] and is essential in placentation and even placental evolution [10]. In early gestation, villi are exposed to low oxygen conditions and express HIF1*α* at high levels. Trophoblasts invade the maternal myometrium and remodel the uterine spiral arteries. Once this process is complete, increased oxygen delivery to the intervillous space results in decreased HIF1*α* expression [8]. However, failed remodeling in individuals with preeclampsia induces the limited dilatation of vessels and strikingly increases the uterine blood velocity, and the latter leads to villus damage and reduces the extraction of oxygen from the intervillous space [11–13], accompanied by persistently elevated HIF1*α* levels in placental tissues [14]. The aforementioned findings indicate that HIF1*α* may mediate the hypoxia adaptation in normal pregnancy and even preeclampsia. However, sustained high expression of HIF1*α* directly causes preeclampsia-like symptoms in animal models [15, 16], indicating the potential pathogenicity of HIF1*α*-induced adaptive responses and suggesting that an adaptation persisting throughout gestation may cause a series of pathological changes both locally in the placenta and peripherally in maternal organs.

However, the mechanism by which the HIF1*α*-related adaptation links placental stress to maternal pathophysiological changes is still unknown. Considering the species specificity of preeclampsia and the high conservation of HIF1*α* in virtually all mammals [17], we speculated that the presence of species-specific molecules may initiate and regulate this mechanism. These molecules should be components of hypoxia-related and even HIF1*α*-regulated, human-specific pathways that connect trophoblast cells and maternal cells.

In the present study, we reported that urothelial cancer associated 1 (UCA1), a hypoxia-responsive long noncoding RNA (lncRNA), was a potentially critical mediator linking the HIF1*α*-induced placental hypoxia adaptation to distal maternal endothelial injury, leading to the occurrence of preeclampsia.

## 2. Results

### 2.1. UCA1 Is a HIF1*α*-Related, Human-Specific lncRNA Involved in Preeclampsia

**Figure 1:**
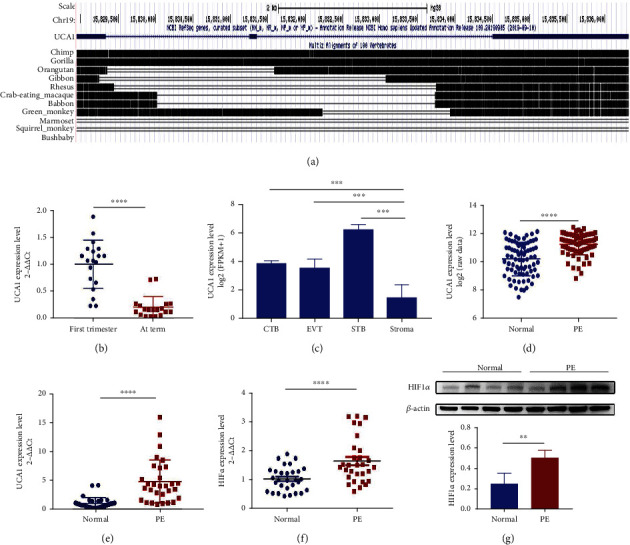
UCA1 is a human-specific lncRNA that is upregulated in preeclampsia. (a) Evolutionary analysis of UCA1 sequences from the UCSC database. (b) The expression level of UCA1 in villous tissues derived from the first trimester and term labor was analyzed using qRT-PCR (*n* =20). (c) Differential expression analysis of publicly available RNA sequencing data from primary trophoblast cells and stromal cells. (d) Differential expression analysis of GSE75010, including preeclamptic placental tissues (*n* =80) and normal controls (*n* =77). (e–f) The RNA levels of UCA1 (e) and HIF1*α* (f) in placental tissues derived from normotensive controls and patients with preeclampsia were analyzed using qRT-PCR (*n* =30). (g) Representative images and statistical analyses of WB showed that HIF1*α* was increased in the preeclamptic placenta. Statistical results were calculated from three independent experiments (*n* =4). ^∗∗^*P* < 0.01, ^∗∗∗^*P* < 0.001, and ^∗∗∗∗^*P* < 0.0001. CTB, cytotrophoblast; EVT, extravillous trophoblast; STB, syncytiotrophoblast; PE, preeclampsia.

As noncoding RNAs are an important part of evolutionary genetics and have been confirmed to play regulatory roles in preeclampsia, seven lncRNAs reported to be associated with hypoxia were selected [6], including UCA1, EFNA3, H19, MALAT1, HOTAIR, FALEC, and HAS2-AS1. An evolutionary analysis of sequences from the UCSC database showed that UCA1 was a human-specific lncRNA. Meanwhile, preeclampsia was also reported in humans and great apes, which showed high UCA1 similarity (Figure 1(a)). The other six lncRNAs did not possess these features (Figure S1).

For a preliminary understanding of the role of UCA1 in normal pregnancy, UCA1 expression levels in placental tissues collected at early and late gestation were measured using qRT-PCR, showing higher UCA1 expression in early pregnancy and attenuated expression in term placentae (Figure 1(b)). An analysis of transcriptome data from primary trophoblast cells that were publicly uploaded by Hiroaki Okae et al. [18] showed that UCA1 has mainly existed in trophoblast cells (Figure 1(c)). A differential expression analysis with a publicly available microarray dataset (GSE75010) was performed to determine the UCA1 expression level in tissues from women with preeclampsia, and the results indicated that UCA1 expression was increased in preeclamptic placentae (Figure 1(d)). Its expression was also confirmed in tissues from our own hospital using qRT-PCR (Figure 1(e)) (30 normotensive pregnant women and 30 preeclamptic women; clinical characteristics were reported in our previous study [19]). Moreover, HIF1*α* was also upregulated in preeclamptic placentae (Figures 1(f) and 1(g)).

### 2.2. HIF1*α* Directly Regulates UCA1 Expression in Trophoblast Cells

**Figure 2:**
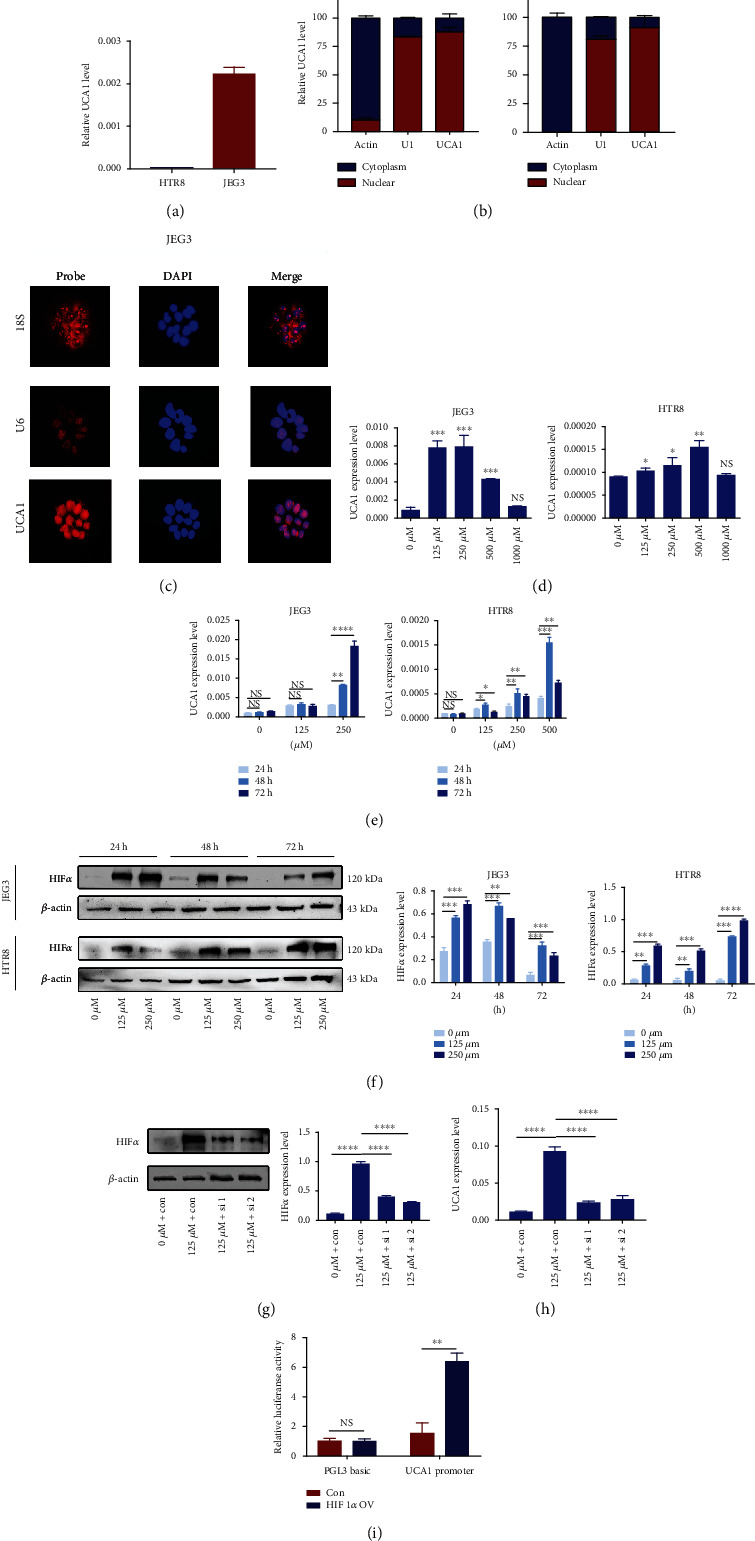
UCA1 is a hypoxia-responsive and HIF1*α*-regulated lncRNA. (a) Endogenous UCA1 expression level in trophoblast cell lines analyzed using qRT-PCR. (b) Subcellular location of UCA1 in both trophoblast cell lines. Nuclear and cytoplasmic RNA was extracted from HTR-8/SVneo and JEG3 cells and differential expression was quantitated by normalization to U1 and actin, respectively, as controls. Data are presented as percentages. (c) RNA fluorescence in situ hybridization of JEG3 cells. Red, RNA probe. Blue, DAPI. Scale bars, 25 *μ*m. (d) Dose-dependent effects of CoCl_2_ on UCA1 expression. (e) Temporal analysis of the effect of CoCl_2_ on UCA1 expression. (f) Representative images and statistical analysis of WB showed that HIF1*α* expression increased with CoCl_2_ treatment, consistent with UCA1 expression. (g) Representative images and statistical analysis of WB showed that the HIF1*α* protein level was decreased after knockdown. (h) The level of UCA1 in cells cultured under hypoxic conditions was also decreased after HIF1*α* knockdown. (i) A dual luciferase reporter assay was conducted following cotransfection with the UCA1 promoter and the HIF-1*α* control and overexpression plasmids. ^∗^*P* < 0.05, ^∗∗^*P* < 0.01, ^∗∗∗^*P* < 0.001, ^∗∗∗∗^*P* < 0.0001, and NS: not significant.

Endogenous expression of UCA1 in HTR-8/SVneo and JEG3 cells was measured to identify the appropriate in vitro models (Figure 2(a)). Both HTR-8/SVneo and JEG3 cell lines were selected for further gain of function experiments, and the JEG3 cell line was used in loss of function assays. We extracted nuclear and cytoplasmic RNA to verify the subcellular localization of UCA1 and found that UCA1 was mainly located in the nucleus rather than in the cytoplasm (Figure 2(b)). The RNA-FISH assay also produced the same results (Figure 2(c)).

CoCl_2_, a compound that induces chemical hypoxia by stabilizing HIF1*α* and 2*α* under normoxic conditions [20], was used to simulate the hypoxia of trophoblast and the increased expression of HIF1*α* in preeclamptic placenta. After an incubation with various concentrations, UCA1 expression exhibited a bidirectional change, increasing significantly after exposure to 125 *μ*M CoCl_2_ to its peak observed in JEG3 cells treated with 250 *μ*M CoCl_2_ and then decreased after treatment with higher concentrations (Figure 2(d)). A similar trend was observed in HTR-8/SVneo cells, with only a slight difference in the peak concentration (500 *μ*M; Figure 2(d)). The temporal analysis showed that after a 48- or 72-hour period, CoCl_2_ treatment effectively increased the UCA1 expression level in both trophoblast cell lines (Figure 2(e)). Hence, concentrations of 250 *μ*M and an incubation time of 48 h were selected for the subsequent establishment of the hypoxia model. In addition, HIF1*α* expression was also tested to evaluate the efficacy of CoCl_2_. As shown in Figure S2, HIF1*α* mRNA levels did not differ, but HIF1*α* protein levels were significantly increased (Figure 2(f)). We also verified that the UCA1 level was significantly decreased after HIF1*α* knockdown under hypoxic conditions (Figures 2(g) and 2(h)). A dual luciferase reporter assay was conducted and verified the direct binding of HIF1*α* to the UCA1 promoter (Figure 2(i)).

### 2.3. UCA1 Plays a Critical Role in the Hypoxic Adaptation of Trophoblast Cells

**Figure 3:**
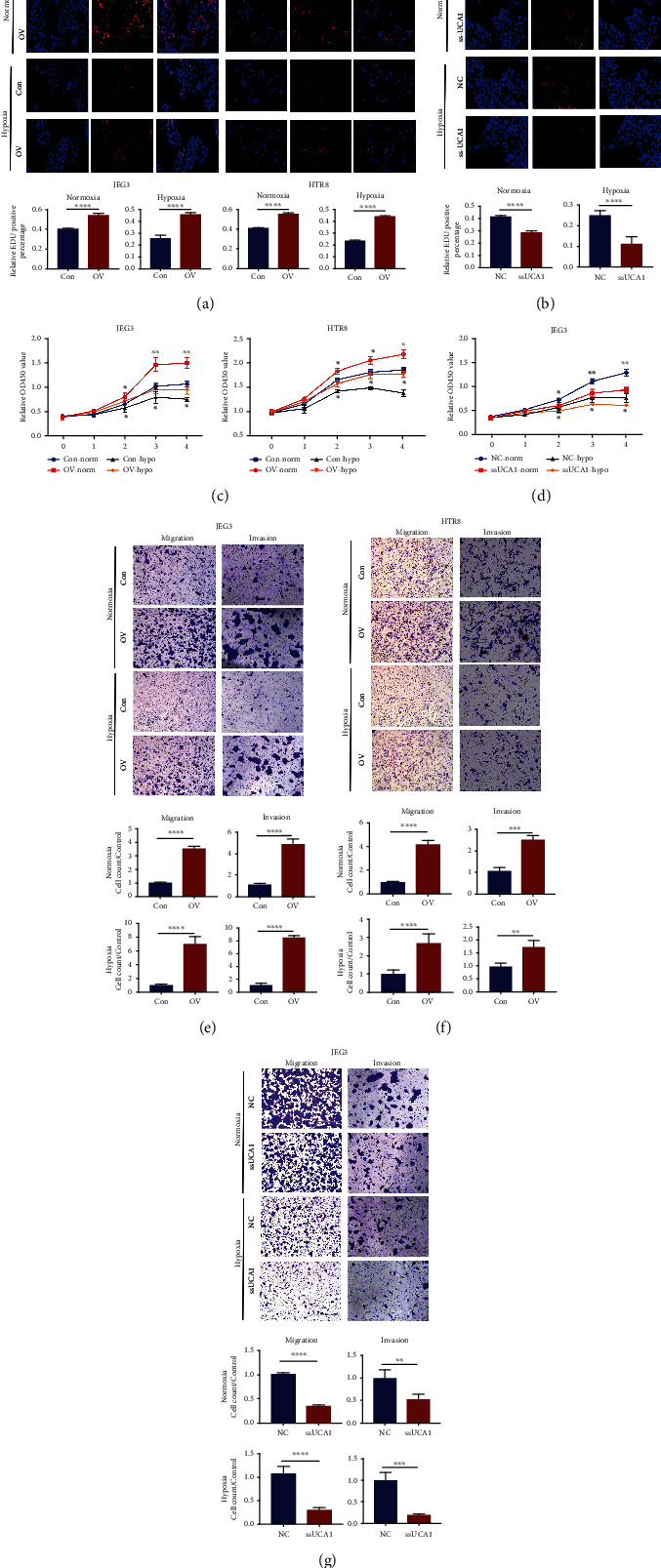
UCA1 increases trophoblast viability under hypoxic conditions. (a–d) EdU (a–b) and CCK8 (c–d) assays of cells with UCA1 overexpression and knockdown. (e–g) Transwell assays of cells with UCA1 overexpression and knockdown. HTR8/SVneo and JEG-3 cells were transfected with UCA1 overexpression and control lentiviruses. JEG-3 cells were transfected with Smart Silencer and negative control. Cells were incubated with 250 *μ*M CoCl_2_ or PBS as a negative control. ^∗∗^*P* < 0.01, ^∗∗∗^*P* < 0.001, and ^∗∗∗∗^*P* < 0.0001.

EdU, Cell Counting Kit-8 (CCK-8), and Transwell assays were performed to evaluate whether UCA1 affects cell proliferation, migration, and invasion capacities under normoxic and hypoxic conditions. Increased UCA1 expression enhanced JEG3 and HTR-8/SVneo cell proliferation (Figures 3(a) and 3(c)) and migration and invasion (Figures 3(e) and 3(f)), and knockdown of endogenous UCA1 significantly suppressed JEG3 cell proliferation (Figures 3(b) and 3(d)) and migration and invasion (Figure 3(g); the transfection efficacy is shown in Figure S3).

**Figure 4:**
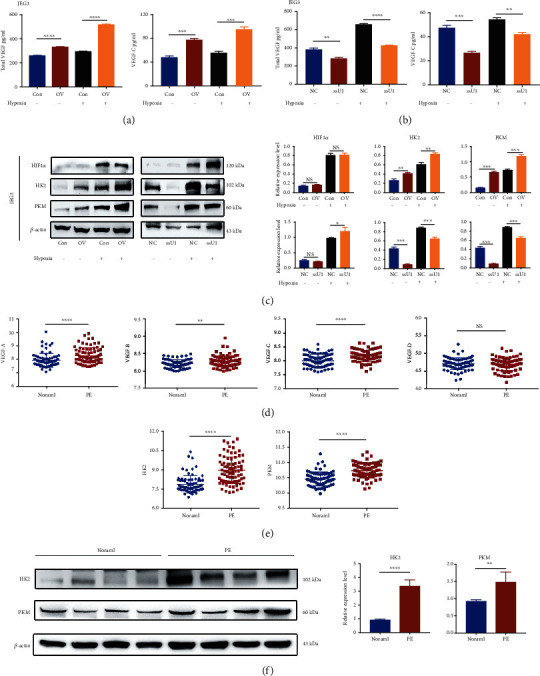
UCA1 promotes VEGF secretion and changes in the levels of key enzymes involved in glycolysis for the hypoxia adaptation. (a–b) Cytokine secretion assays of cells with UCA1 overexpression and knockdown. (c) Representative images and statistical analysis of WB showed that UCA1 overexpression increased the levels of HK2 and PKM, while UCA1 knockdown decreased their expression levels. (a–c) JEG-3 cells were transfected with the UCA1 overexpression plasmid, Smart Silencer, and their negative controls. Cells were incubated with 250 *μ*M CoCl_2_ or PBS as a negative control. (d–e) Differential expression analysis of VEGF-A/B/C/D (d), HK2 and PKM (e) in preeclamptic placentae (*n* =80) and normal controls (*n* =77) from GSE75010. Data were calculated as log2 (row data). (f) Representative images and statistical analysis of WB showed that HK2 and PKM levels were increased in preeclamptic placentae. ^∗^*P* < 0.05, ^∗∗^*P* < 0.01, ^∗∗∗^*P* < 0.001, ^∗∗∗∗^*P* < 0.0001, and NS: not significant.

As reported, HIF1*α* is a main regulator of vascular endothelial growth factor (VEGF) production and cell metabolism in the hypoxia adaptation; the latter involves shifting metabolism to glycolysis during hypoxia. We measured the levels of secreted cytokines and key glycolytic enzymes, including hexokinase 2 (HK2) and pyruvate kinase M1/2 (PKM), to investigate the regulatory effect of UCA1 on cellular secretion and glycolysis. The upregulation of UCA1 in JEG3 cells promoted the secretion of total VEGF and VEGF-C under both normoxic and hypoxic conditions (Figure 4(a)), while cells with UCA1 knockdown secreted less VEGF (Figure 4(b)). Moreover, HK2 and PKM levels were increased upon UCA1 upregulation and decreased upon UCA1 knockdown in JEG3 cells (Figure 4(c)). Then, we analyzed the GSE75010 dataset and found that the mRNA levels of VEGF, including VEGF-A/B/C but not VEGF-D, were increased in preeclamptic placentae (Figure 4(d)), similar to HK2 and PKM (Figure 4(e)). The HK2 and PKM protein levels were also increased in preeclamptic placentae, as confirmed by western blotting (Figure 4(f)). Considering the importance of VEGF in angiogenesis and glycolysis in glucose metabolism, these findings indicated that UCA1 may play a critical role in promoting the cellular adaptation and survival in hostile environments, such as hypoxia.

### 2.4. Exosomal UCA1 Is Upregulated in Maternal Serum from Patients with Preeclampsia and Supernatants of Trophoblast Cells under Hypoxic Conditions

**Figure 5:**
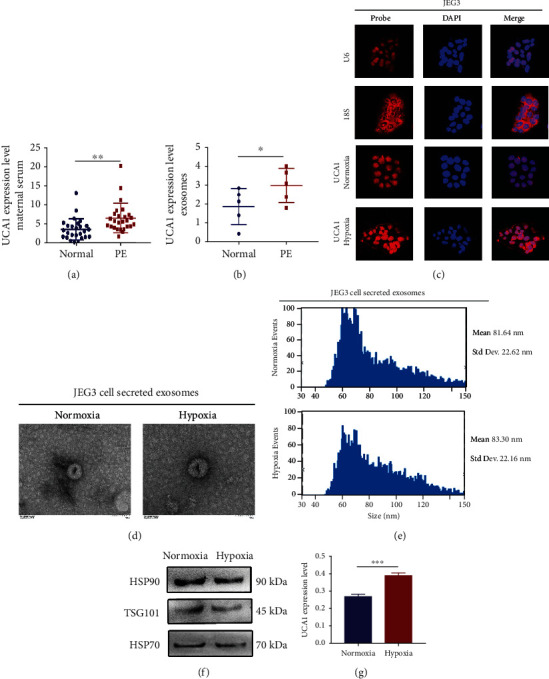
Circulating exosomal UCA1 levels are increased in maternal serum from patients with preeclampsia and supernatants of hypoxic trophoblast cells. (a) UCA1 expression level in maternal serum derived from normotensive pregnant women and patients with preeclampsia, as determined using qRT-PCR (*n* =20). (b) UCA1 expression level in serum exosomes extracted from normotensive pregnant women and patients with preeclampsia determined using qRT-PCR (*n* =5). (c) Fluorescence images showing the nuclear export of UCA1 in JEG-3 cells under hypoxic conditions. Red, RNA probe. Blue, DAPI. Scale bars, 25 *μ*m. (d) TEM images showing exosomes derived from the supernatant of trophoblasts. Cells were incubated with 250 *μ*M CoCl_2_ or PBS as a negative control. Scale bars, 100 nm. (e) Exosome NTA showing the average size of exosomes derived from the supernatant of trophoblasts. (f) Representative images of WB for the exosomal markers HSP90, HSP70, and TSG101 in exosomes derived from the supernatant of trophoblasts. (g) UCA1 expression level in exosomes extracted from the supernatant of trophoblasts using qRT-PCR. Cells were incubated with 250 *μ*M CoCl_2_ or PBS as a negative control. ^∗^*P* < 0.05, ^∗∗^*P* < 0.01, and ^∗∗∗^*P* < 0.001.

Cell-free UCA1 levels were measured in maternal serum from patients with preeclampsia using RT-qPCR to investigate whether UCA1 is the potential link between placenta stress and maternal injury, and the results showed that UCA1 expression was increased in patients with preeclampsia (Figure 5(a)) (20 normotensive pregnant women and 20 preeclamptic women; the clinical characteristics of the patients are listed in Table S2.). Considering the instability of cell-free lncRNAs and the stable existence of UCA1 in exosomes reported in previous studies on cancers [21–23], serum exosomal UCA1 levels were measured and were higher in patients with preeclampsia (Figure 5(b)).

RNA-FISH of JEG3 cells under hypoxic conditions was repeated and showed that a portion of UCA1 translocated to the cytoplasm under hypoxic conditions, explaining how nuclear-located UCA1 was packed into exosomes that are mainly generated in the cytoplasm (Figure 5(c)). Transmission electron microscopy (TEM) showed a similar morphology and round-shaped appearance in both JEG3-derived normoxic and hypoxic exosomes (Figure 5(d)). Nanoparticle tracking analysis (NTA) presented a similar distribution in both groups (Figure 5(e)). The levels of three well-known exosomal markers, TSG101, HSP70, and HSP90, were confirmed by western blotting (Figure 5(f)). The results described above indicated the success of exosome isolation. Then, the exosomal UCA1 expression level was detected using RT-qPCR, and it was upregulated in the hypoxic cellular supernatant (Figure 5(g)). Hence, exosomal UCA1 expression was increased in the maternal circulation of women with preeclampsia and the supernatant of hypoxic trophoblast cells, implying that UCA1 might be a mediator connecting trophoblasts and maternal peripheral cells.

### 2.5. Hypoxic Trophoblast Cell-Derived Exosomes Induce ROS Production in HUVECs

**Figure 6:**
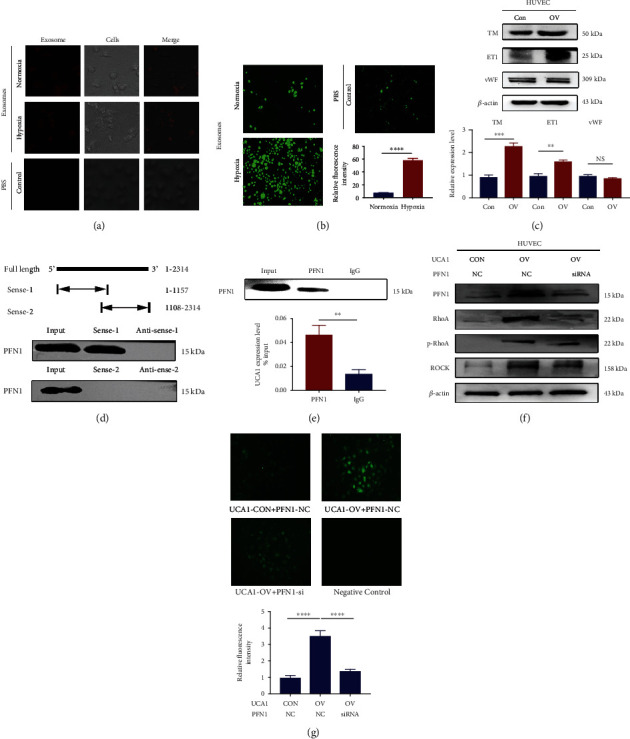
Exosomal UCA1 promotes endothelial injury in HUVECs by inducing ROS production. (a) Fluorescence images showing absorbed exosomes in HUVECs. Exosomes were derived from the supernatant of trophoblasts incubated under normoxic and hypoxic conditions. Red, exosomes labeled with the Exo-Red dye. PBS was used as a negative control. Scale bars, 25 *μ*m. (b) Fluorescence images showing ROS production in HUVECs incubated with exosomes. Scale bars, 300 *μ*m. (c) Representative images and statistical analysis of WB showing that the overexpression of UCA1 increased the expression of some markers of endothelial injury, including TM and ET1. (d) RNA pulldown assay showing the binding of PFN1 to UCA1 truncation (1-1157), rather than the other truncation (1108-2314). (e) The RIP assay also showed that UCA1 bound to PFN1. (f) Representative WB images showing that UCA1 overexpression increased the expression of PFN1 and critical molecules in the RhoA/ROCK pathways; knockdown of PFN1 inhibited the UCA1-induced activation of the RhoA/ROCK pathway. (g) Fluorescence images showing that UCA1 overexpression significantly increased ROS production in HUVECs, while PFN1 knockdown inhibited UCA1-induced ROS production. HUVECs were transfected with UCA1 overexpression and control lentiviruses and PFN1-siRNA and control siRNA. ^∗∗^*P* < 0.01, ^∗∗∗^*P* < 0.001, ^∗∗∗∗^*P* < 0.0001, and NS: not significant.

As maternal endothelial dysfunction and oxidative stress are the leading pathophysiological features of preeclampsia, we explored whether hypoxic exosomes derived from trophoblast cells induce endothelial injury, especially ROS production. As shown in Figure 6(a), Exo-Red dye-labeled exosomes were successfully internalized by HUVECs. Moreover, in Figure 6(b), ROS production was significantly increased in HUVECs incubated with hypoxic exosomes compared with normoxic exosomes. Upregulation of UCA1 also increased the levels of thrombomodulin (TM) and endothelin (ET) 1, which are markers of the injured vessel endothelium, but not von Willebrand factor (vWF) (Figure 6(c)), indicating the potential endothelial injury induced by UCA1.

As a method to further explore the mechanism, we performed an RNA pulldown assay combined with mass spectrometry (MS) to identify the proteins interacting with UCA1 and focused on profilin 1 (PFN1). This protein is reported to play an important role in regulating actin polymerization, reorganization, and redistribution of the cytoskeleton, forming a pathological basis for endothelial cell contraction and contributing to endothelial abnormalities and vascular diseases [24–26]. Western blot assays showed that the PFN1 protein was enriched in the pulldown products of truncated UCA1 (1-1157) compared with the antisense RNA. Another truncation (1108-2314) did not precipitate any protein, suggesting that PFN1 might interact with UCA1 at a site between nucleotides 1-1108 (Figure 6(d)). RNA-binding protein immunoprecipitation (RIP) assays were also performed to verify the interaction between UCA1 and PFN1 (Figure 6(e)). These data confirmed the interaction between UCA1 and PFN1 at the structural level.

An RNA-seq analysis of HUVECs with or without UCA1 overexpression was conducted to further identify related signaling pathways, and 606 differentially expressed genes (DEGs) were identified (Figure S4a). According to the KEGG database, we focused on the pathway of regulation of the actin cytoskeleton (Figure S4b), which is closely related to cellular morphological changes, migration, and contraction, and even endothelial injury [26]. Key molecules involved in this pathway were further validated by performing western blot analyses, including PFN1, RhoA, p-RhoA, and Rho-kinase (ROCK). The results suggested that the RhoA/ROCK pathway was activated in HUVECs after UCA1 overexpression and was inhibited after PFN1 knockdown (Figure 6(f)). Then, we also analyzed ROS production and found that UCA1 overexpression significantly induced ROS production, while this auxo-action was inhibited by PFN1 knockdown (Figure 6(g)).

### 2.6. UCA1 Functions as a Scaffold for USP14 and PFN1 to Prolong the Half-Life of the PFN1 Protein

**Figure 7:**
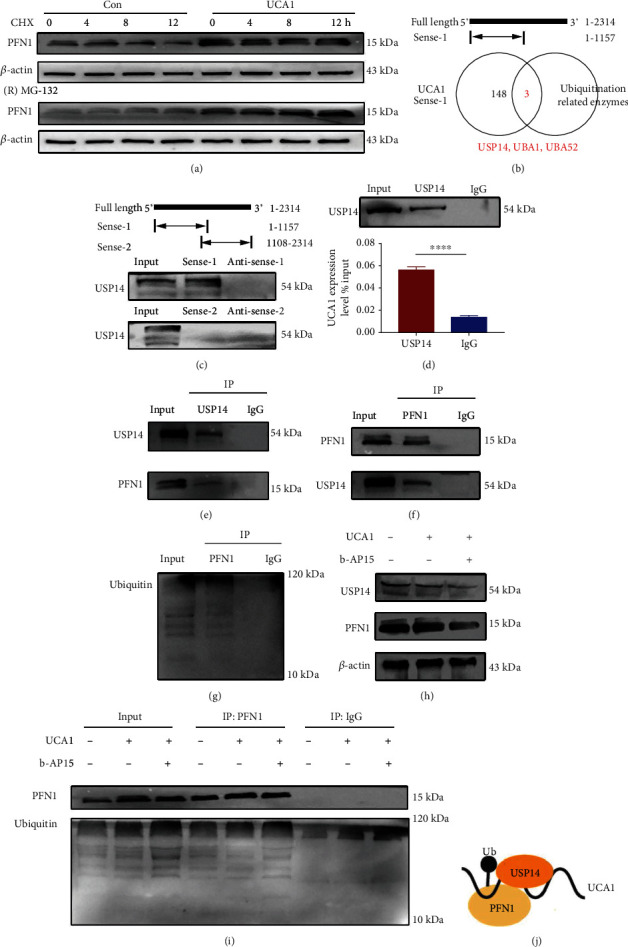
UCA1 functions as a scaffold for USP14 and PFN1 to prolong the half-life of the PFN1 protein by modulating ubiquitination. (a) HUVECs transfected with UCA1 overexpression and control vectors were treated with CHX (50 *μ*g/ml) and (R)-MG132 (20 *μ*M) for the indicated times, respectively. PFN1 protein levels were measured using WB. (b) Venn diagram showing that three ubiquitination-related enzymes were enriched in the pulldown products of truncated UCA1 (1-1157), which was consistent with PFN1. (c) RNA pulldown assay showing the binding of USP14 to truncated UCA1 (1-1157). (d) The RIP assay also showed the specific interaction between UCA1 and USP14. (e) A Co-IP assay was performed for USP14 and PFN1. (f, g) Co-IP assays were performed for PFN1 and USP14, and PFN1 and ubiquitin. (h) HUVECs transfected with UCA1 overexpression and control vectors were treated with b-AP15 (100 nM) for 3 h. USP14 and PFN1 protein levels were measured using WB. (i) The Co-IP assay showed that the inhibition of USP14 increased the level of ubiquitinated PFN1. (j) UCA1 functions as a scaffold for USP14 and PFN1.

We measured the half-life of the PFN1 protein to investigate the underlying mechanism by which UCA1 regulates the PFN1 protein. Cells were treated with the protein synthesis inhibitor cycloheximide (CHX) and the proteasome inhibitor (R)-MG-132. UCA1 prolonged the half-life of the PFN1 protein (Figure 7(a)). Previous studies have reported that UCA1 regulates ubiquitination-dependent degradation [27, 28] and verified the existence of proteasome-dependent PFN1 degradation [29, 30]. Hence, we hypothesized that UCA1 might function as a scaffold to recruit ubiquitination-related enzymes and PFN1, further regulating PFN1 ubiquitination-dependent degradation. According to the MS data from the UCA1 RNA pulldown, three ubiquitination-related enzymes were enriched in the pulldown products with truncated UCA1 (1-1157), which was consistent with PFN1, including USP14, ubiquitin-like modifier activating enzyme 1 (UBA1), and UBA52 (Figure 7(b)). Among them, USP14 is a member of the ubiquitin-specific processing family of proteases and functions as a deubiquitinating enzyme. The RNA pulldown immunoblot analysis showed the specific association between UCA1 and USP14, indicating the potential existence of a ternary complex (Figure 7(c)). Using RIP and Co-IP assays with an anti-USP14 antibody, we detected that USP14 interacted with UCA1 and PFN1, respectively (Figures 7(d) and 7(e)). Then, another Co-IP assay with an anti-PFN1 antibody was performed and showed that PFN1 bound to USP14 and ubiquitin (Figures 7(f) and 7(g)). In addition, we used the USP14 inhibitor b-AP15 to verify the regulation of USP14 in this ternary complex. The inhibition of USP14 promoted PFN1 degradation (Figure 7(h)) by increasing the ubiquitination of PFN1 (Figure 7(i)). Therefore, we verified that UCA1 functioned as a scaffold for USP14 and PFN1 to regulate the ubiquitination-dependent degradation of PFN1 (Figure 7(j)). In summary, UCA1 may physically function as a scaffold for USP14 to PFN1 to prolong the half-life of the PNF1 protein, subsequently activate the RhoA/ROCK pathway, promote ROS production, and induce endothelial injury.

## 3. Discussion

**Figure 8:**
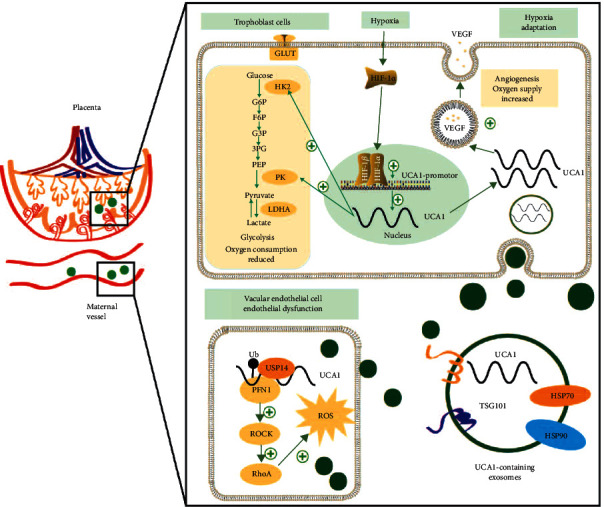
Graphic showing the mechanism of the human-specific UCA1-mediated adaptive response in preeclampsia. Because of failed remodeling, placental stress, and hypoxia, trophoblasts express HIF1*α* at high levels, which binds to the UCA1 promoter to upregulate its expression. In trophoblasts, UCA1 promotes the hypoxia adaptation by increasing VEGF secretion and the expression levels of PKM and HK2, both of which are key enzymes involved in glycolysis. Cytoplasmic UCA1 is packed into exosomes and transferred to vascular endothelial cells. In endothelial cells, UCA1 functions as a scaffold to recruit USP14 and PFN1, and prolong the half-life of the PFN1 protein, subsequently activating the RhoA/ROCK pathway to produce excess ROS and induce endothelial injury. In mothers, HIF1*α*-UCA1-induced adaptive responses may induce the clinical symptoms of preeclampsia.

Preeclampsia is a species-specific disease related to placental evolution and the HIF1*α*-induced hypoxia adaptation. UCA1 is a human-specific lncRNA regulated by HIF1*α* and is involved in the pathogenesis of preeclampsia. UCA1 promoted the local hypoxia adaptation of trophoblast cells and linked placental stress to endothelial injury through circulating exosomes. At the molecular level, UCA1 increased VEGF secretion and the expression levels of key enzymes involved in glycolysis to allow the cells adapt to placental hypoxia. In endothelial cells, UCA1 formed a ternary complex with USP14 and PFN1, prolonged the half-life of the PFN1 protein, and subsequently activated the RhoA/ROCK pathway to produce excess ROS and induce endothelial dysfunction (Figure 8). Therefore, this study provides an evolutionary perspective and insights into the pathogenesis of preeclampsia, namely, HIF1*α*/UCA1-induced adaptive responses that link the placental hypoxia adaptation to distant endothelial injury.

HIF1*α* is a critical and adaptive molecule involved in normal gestation, and its expression in human placentae is increased in the first trimester and then decreases at 9 weeks because of the increase in circulation and oxygenation [8]. Persistently increased HIF1*α* expression was observed in preeclamptic placentae; overexpression of HIF1*α* in pregnant animals resulted in several symptoms of preeclampsia [15, 16]. Thus, HIF1*α* is considered a pathogenic molecule involved in preeclampsia. In the present study, the expression of human-specific UCA1 showed the same change as HIF1*α* expression in normal pregnancy and preeclampsia: high expression in early gestation that dwindled during normal pregnancy but was upregulated in the preeclamptic placenta again. Then, UCA1 was confirmed to be a hypoxia-responsive and HIF1*α*-regulated lncRNA, similar to other studies in cancers [31, 32], suggesting that UCA1 is a downstream molecule of the HIF1*α* pathway and hypoxia responses in women with preeclampsia.

Our findings indicated that UCA1 may have two-way functions in different cells. On the one hand, UCA1 responded to hypoxia and induced the hypoxic adaptation in trophoblast cells, including the activation of cellular biofunctions, glycolysis, and VEGF secretion. This function of UCA1 was consistent with that reported in previous studies of UCA1-induced cellular adaptation and survival, including proerythroblast differentiation [33], adaptation to a toxic environment [34], and tumor progression [35, 36]. Clara Apicella et al. [37] explored the role of UCA1 in activating syncytiotrophoblast, and they also speculated that the upregulation of UCA1 in the preeclamptic placenta might reflect an increase in the turnover of the syncytium to compensate for an increased apoptotic rate. The study by Hongfang Shao et al. [38] also supports the positive regulation of UCA1 in trophoblast invasion, especially in the first trimester. Based on this knowledge, we inferred that the elevated UCA1 expression in preeclampsia represents an incomplete compensatory mechanism secondary to placental ischemia and hypoxia instead of the cause of impaired trophoblast biofunction.

On the other hand, in endothelial cells, we found that trophoblast-derived hypoxic exosomes and UCA1 induced ROS production and endothelial injury through the PFN1-RhoA/ROCK pathway. The systemic presentations of preeclampsia arise from factors released from the stressed placentae, such as hypoxia-induced soluble Fms-like (sFLT)-1 [39]. Serous exosomes were also reported to induce preeclampsia by delivering sFlt-1 and soluble endoglin (sEng) or other constituents of the complex [40–43]. According to our results, UCA1-containing exosomes acted as mediators between hypoxic trophoblasts and vascular endothelial cells. More specifically, exosomal UCA1 induced endothelial injury by promoting ROS production. Moreover, a molecular relationship among UCA1, USP14, and PFN1 and the downstream RhoA/ROCK pathway was confirmed. PFN1 has been reported to regulate the reorganization and redistribution of the cytoskeleton to mediate endothelial contraction, which results in vascular hyperpermeability; it also activates RhoA/ROCK signaling to produce ROS. This regulation was similar to the role of UCA1 in sepsis [44, 45] and neurological injury [46]. Therefore, we assumed that UCA1 may have dual functions in preeclampsia and other complex pathological environments. In our opinion, the different biofunctions of UCA1 are more likely to be the regulatory strategy for adaptation.

**Figure 9:**
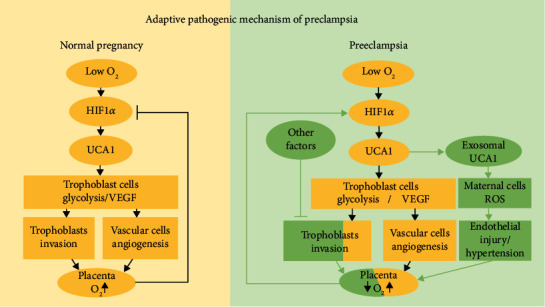
Graphic presenting the theory of HIF1*α*-induced adaptive responses in normal pregnancy and preeclampsia. (Left panel) In early gestation, trophoblasts are exposed to low oxygen levels and express HIF1*α* and UCA1 at high levels to regulate the trophoblast adaptation to hypoxia, including promoting glycolysis to reduce oxygen consumption and secreting VEGF to induce angiogenesis. With the deep invasion of trophoblasts and remodeling of spiral arteries, dilatation reduced the velocity of maternal blood into the intervillous space, and oxygen and nutrients are fully exchanged. Hence, HIF1*α* and UCA1 levels gradually decrease; this negative feedback loop is attenuated with the increase in gestational weeks in a normal pregnancy. (Right panel) With impaired trophoblast invasion and failed vessel remodeling caused by other factors, the transfer of oxygen and nutrients to the fetus and villous tree is decreased, leading to hypoxic and stressed placental conditions. The trophoblast cells express HIF1*α* and UCA1 at high levels again, promoting the local adaptation to hypoxia. However, these strategies do not completely compensate for sustained local hypoxia, and thus, trophoblasts secrete UCA1-containing exosomes that are transported to distant maternal cells and cause peripheral changes, including a series of manifestations characterized by hypertension, to partially relieve placental ischemia and hypoxia.

Based on these findings, we developed a novel theory of preeclampsia, namely, a HIF1*α*-induced adaptive mechanism. From the perspective of the fetus and placentae, when deep invasion and spiral artery remodeling are impaired by other unknown factors, trophoblasts are under persistent hypoxia; hence, UCA1 is upregulated to promote the hypoxia adaptation, but this compensatory mechanism is incomplete. Hypoxic trophoblasts have no alternative but to secrete exosomal UCA1 to induce endothelial injuries, which further attempts to increase maternal blood pressure and relieve placental ischemia as a mechanism to partially compensate for the insufficiency of trophoblasts (Figure 9). Finally, the dual functions of UCA1 in different cell types should be discussed. This adaptive mechanism is actually a fetal-led survival strategy that benefits itself but harms the mother, which might fully explain the clinical phenomena observed in women with preeclampsia, namely, maternal hypertension improves placental ischemia and hypoxia, and antihypertensive treatment exerts the opposite effect.

Some limitations are worth noting. First, chemical hypoxia does not simulate placental conditions in vivo, but it is consistent by inducing high expression of HIF1*α*; then, appropriate concentrations were also selected to reduce the side effects [47–49]. Second, we did not construct UCA1-rich exosomes to evaluate their potential effect on HUVECs, and thus, other constituents may also cause endothelial injury. In the present study, UCA1 was overexpressed in HUVECs, and the results, including increased levels of endothelial injury markers and elevated ROS production, were consistent with those obtained from cells incubated with UCA1-containing hypoxic exosomes. Nevertheless, further exploration of other exosomal molecules from trophoblast cells, especially those with species specificity, is necessary. Third, we only explored the effect of exosomal UCA1 on endothelial cells but not proteinuria and coagulation disorders; further studies are needed to determine the potential connection among these processes. Fourth, we did not have appropriate animal models to confirm this mechanism partially because of the low homology in mice or rats. Therefore, more appropriate models may need to be explored, such as primate models and organoids [50, 51].

## 4. Conclusions

This study proposes a novel theory to elucidate how placental dysfunction may cause pathophysiological features of preeclampsia, namely, the human-specific UCA1-mediated adaptive responses. Further studies of these primate-specific molecules may help to elucidate the pathogenesis of evolution-related diseases, including but not limited to preeclampsia, diabetes mellitus, obesity, cardiovascular diseases, and tumors.

## 5. Methods

### 5.1. Ethics Statement and Clinical Specimens

All participants provided written informed consent, and the study was approved by the ethics committee of the Obstetrics and Gynecology Hospital of Fudan University (2019-124). Human placental tissues were divided into three groups: villous tissues from women in early gestation (6-8 gestational weeks, normal patients undergoing abortion, *n* =20), placental tissues from women with preeclampsia (*n* =30), and those from normotensive pregnant women (*n* =30). Placental tissues were collected after delivery. Maternal serum was also collected from women with preeclampsia and normotensive pregnant women (*n* =20). Approximately 5 ml of venous blood were collected before cesarean delivery. The blood was collected into coagulation-promoting tubes, and the upper layer containing maternal serum was collected after 3 minutes of standing. Preeclampsia was diagnosed according to the ACOG Guidelines [52].

The clinical characteristics of women with preeclamptic and normotensive pregnant women are described in our previous study [19]. In addition, Table S1 shows the maternal age and pre-gestational BMI of women in early gestation and women with a full-term pregnancy who had a normal blood pressure. The clinical characteristics of preeclamptic women and normotensive pregnant women who provided the maternal peripheral blood samples before delivery are shown in Table S2, and maternal blood pressure and birth weight were also adjusted for maternal age, pre-gestational BMI, and gestational age at delivery.

### 5.2. Differential Expression Analysis of UCA1

The RNA-seq data from primary trophoblast cells and stromal cells were downloaded from the sequencing files submitted by Hiroaki Okae et al. [18]. The UCA1 expression level is reported as log2 (FPKM+1) in Figure 1(c). A publicly available RNA microarray dataset (GSE75010, https://www.ncbi.nlm. http://nih.gov/geo/query/acc.cgi?acc=GSE75010) was downloaded from the GEO database and used to evaluate UCA1 expression in human placental tissues from women with preeclampsia (*n* =80) and normotensive pregnancies (*n* =77). After normalization and log2 transformation of the raw data, the comparison of the relative expression level of UCA1 between the two groups is presented in Figure 1(d).

### 5.3. Total RNA Extraction and qRT-PCR

Total RNA was extracted using TRIzol reagent (Invitrogen) according to the manufacturer's instructions. RNA was reverse transcribed to cDNAs using a PrimeScript RT Reagent Kit (TaKaRa). A SYBR Green qPCR assay (Takara) and gene-specific primers were used for qRT-PCR; *β*-actin was used for normalization. The relative expression levels of genes were calculated using the 2^−*ΔΔ*Ct^ method. The primer sequences are shown in Table S3.

### 5.4. Cell Culture

JEG3 cells were obtained from the Cell Bank of the Chinese Academy of Science (Shanghai, China). HUVECs and HEK293T cells were gifts from the School of Basic Medical Sciences, Fudan University (China). All cells were cultured in DMEM (Biological Industries). HTR-8/SVneo cells were a kind gift from Dr. Charles Graham (Queens University, Kingston, ON, Canada) and were cultured in RPMI-1640 medium (Biological Industries). Complete medium was supplemented with 10% fetal bovine serum (FBS, Gibco; Thermo Fisher Scientific), 100 U/ml penicillin, and 100 *μ*g/ml streptomycin (Invitrogen).

For hypoxia experiments, hypoxic conditions were induced using CoCl_2_-6H_2_O (Sigma). Our use of CoCl_2_ was mainly based on both placental hypoxia and high expression of HIF1*α* and 2*α* in women with preeclampsia. More specifically, cells were incubated with various concentrations of CoCl_2_ (0 *μ*M, 125 *μ*M, 250 *μ*M, 500 *μ*M, or 1000 *μ*M) or with 250 *μ*M CoCl_2_ for various periods (24 h, 48 h, or 72 h). CoCl_2_ may cause biobehavioral changes in trophoblasts, such as cellular senescence and death. Finally, an incubation with 250 *μ*M CoCl_2_ for 48 h was selected for subsequent experiments.

### 5.5. Nuclear and Cytoplasmic Extraction

Cytoplasmic extraction buffer (0.5 ml; Invent Biotechnologies) was added to the cells and incubated on ice for 5 min. Lysates were transferred to tubes and mixed vigorously for 15 sec. Then, lysates were centrifuged at 12,000 × g for 4 min at 4°C, and TRIzol was added to the nuclear (pellet) and cytoplasmic (supernatant) fractions for RNA extraction. Additional steps are described in part 2.

### 5.6. RNA Fluorescence In Situ Hybridization (RNA-FISH)

This assay was performed with a FISH kit (Guangzhou RiboBio Co., Ltd.) according to the manufacturer's instructions. Briefly, fixed cells were further incubated with 20 *μ*M FISH probe in hybridization buffer (RiboBio) at 37°C overnight. Then, the cells were stained with DAPI, and fluorescence signals were detected using a fluorescence microscope.

### 5.7. Luciferase Reporter Assay

293T cells were transfected with luciferase reporter plasmids containing the 3′ untranslated region (UTR) of HIF1*α* or the UCA1 promoter (both constructed by Generay Biotech Co., Ltd.) using Lipofectamine 3000 according to the manufacturer's instructions. After 48 h, luciferase activity (Dual Luciferase Reporter Gene Assay Kit; Promega) was measured using a microplate reader, and Renilla luciferase activity was used for normalization.

### 5.8. Plasmid Construction and Cell Transfection

For UCA1 overexpression, the full-length human UCA1 sequence (2314 bp) was cloned into the pGMLV-CMV-MCS-PGK-Puro vector to construct the UCA1 overexpression vector (Genomeditech, Shanghai, China).

Considering the nuclear localization of UCA1, we used lncRNA Smart Silencer (Guangzhou RiboBio Co., Ltd.), a mixture of three antisense oligonucleotides and three siRNAs, according to the manufacturer's instructions. For HIF1*α* and PFN1 knockdown, 50 nM siRNA was transfected into cells using Lipofectamine 3000 transfection reagent. The knockdown efficiency was validated using qRT-PCR and western blotting.

### 5.9. Cell Proliferation, Migration, and Invasion Assays

Cell proliferation and viability were assessed using EdU (Guangzhou RiboBio Co., Ltd.) and CCK8 assays (Yeasen Biotechnology Co., Ltd.). Cell migration and invasion were evaluated using Transwell chambers with 8-*μ*m pore size inserts (Corning).

### 5.10. Analysis of Cytokine Levels in the Cell Supernatant

The levels of cytokines in cell supernatants were measured using a human cytokine magnetic bead panel (Millipore, Billerica, MA, USA). The supernatant samples were assayed in triplicate, and the average concentration of each cytokine was calculated. The cytokine concentrations were measured according to the manufacturer's protocol and eventually analyzed using a Luminex 200 analyzer (Luminex, Austin, TX, USA).

### 5.11. Western Blotting (WB)

Cells were lysed, and total protein was extracted using RIPA lysis buffer (Beyotime Institute of Biotechnology) supplemented with protease inhibitors. The protein concentration was normalized using the Bradford method for relative protein quantification. Proteins were separated on sodium dodecyl sulfate-polyacrylamide gel electrophoresis (SDS-PAGE) gels (New Cell & Molecular Biotech Co., Ltd) and transferred to a nitrocellulose membrane. The membrane was incubated with a specific primary antibody. Horseradish peroxidase-conjugated secondary antibodies were used. *β*-Actin served as the control. Protein expression was assessed with ECL reagents. The details of the antibodies used in this study are shown in Table S4.

### 5.12. Total Serum RNA Extraction and Exosome Isolation

Total RNA was extracted from maternal serum using a BIOG cfRNA Easy Kit (Changzhou Biogenerating Biotechnology Corp.) according to the manufacturer's protocol. Exosomes were isolated from maternal serum samples using ExoQuick solution according to the manufacturer's instructions (System Biosciences, SBI, Mountain View, CA). Total exosomes from cell culture media were isolated using Total Exosome Isolation Reagent (Thermo Fisher Scientific, Inc.) according to the manufacturer's instructions. JEG3 cells were cultured in DMEM without FBS under normoxic or hypoxic conditions for 48 h. Exosome pellets were resuspended in PBS.

### 5.13. Transmission Electron Microscopy (TEM) and Nanoparticle Tracking Analysis (NTA)

According to the TEM sample preparation procedure, the exosome suspension was dripped on a copper grid placed on filter paper and dried. Next, the exosomes were stained with one drop of uranyl acetate. The samples were dried for 10 min at room temperature. The exosomes were visualized using a TEM (HT7700, Hitachi, Tokyo, Japan) at 100 keV. The size distribution of exosomes was detected using a NanoFCM N30E according to the manufacturer's instructions. Briefly, the exosome suspension was filtered with a syringe filter (Millipore). Then, the samples were diluted until the individual nanoparticles were detected, and the size distribution was evaluated.

### 5.14. Exosome Labeling

Exosomes derived from normoxic or hypoxic cell media were labeled with the ExoGlow™-Membrane EV Labeling Kit (SBI) according to the manufacturer's instructions. HUVECs were incubated with labeled exosomes for 6 h at 37°C.

### 5.15. Intracellular ROS Measurement

Intracellular ROS levels were detected using a 2′,7′-dichlorofluorescin diacetate (DCFDA)-cellular ROS assay kit (Abcam). After an incubation with labeled exosomes or transfection, the cell culture media was removed. HUVECs were stained with 20 *μ*M DCFDA for 30 min at 37°C. Images were captured using a fluorescence microscope.

### 5.16. RNA Pulldown Assay

UCA1 and antisense RNA were transcribed in vitro with Taq Master Mix (Vazyme, China). Then, the products were labeled with T7 biotin using T7 polymerase and biotin RNA labeling mix. Biotinylated RNAs were incubated with cell lysates and magnetic beads for 1 h. The RNA-protein complex was boiled in loading buffer and subsequently analyzed using mass spectrometry (LC Sciences, China) and WB assays. The primer sequences used to amplify the template DNA are listed in Table S3.

### 5.17. RNA Immunoprecipitation (RIP)

Briefly, 4 × 10^7^ cells were harvested and resuspended in 200 *μ*l of lysis buffer. The samples were incubated overnight at -80°C. Protein A/G magnetic beads (Thermo Fisher Scientific, Inc.) were washed twice with dilution buffer and incubated with 5 *μ*g of anti-PFN1 or rabbit IgG antibody for 1 h at room temperature. The beads were washed five times with dilution buffer and resuspended in dilution buffer. One hundred microliters of samples was added to the beads and the mixture was incubated overnight at 4°C. The beads were washed five times with dilution buffer again, and RNA-antibody bead complexes were digested with 1.2 mg/ml Proteinase K for 30 min at 55°C. Finally, RNA was extracted with TRIzol and measured using qRT-PCR.

### 5.18. Protein Half-Life Analyses

For the protein half-life analysis, cells were incubated with 50 *μ*g/ml cycloheximide (MedChemExpress LLC) for 0, 4, 8, or 12 h, and then, protein levels were measured using WB. In addition, 20 *μ*M (R)-MG132 (MedChemExpress LLC) was used to effectively inhibit the proteasome, and the protein levels were also measured using WB.

### 5.19. Coimmunoprecipitation (Co-IP)

Briefly, 1 × 10^7^ cells were lysed using immunoprecipitation assay buffer (New Cell & Molecular Biotech Co., Ltd) for 30 min on ice. Then, the lysates were centrifuged at 12000 rpm for 15 min, and the supernatant was incubated with 20 *μ*l of protein A/G-agarose beads and the indicated antibodies at 4°C overnight. Afterward, the beads were washed with NP40 buffer three times, followed by WB assays.

### 5.20. Statistical Analysis

Statistical analyses of clinical samples were performed using SPSS version 20.0 (SPSS Inc., IL, USA), and analyses of experimental results were carried out using GraphPad Prism 7 software (GraphPad, USA). The differences between two groups were evaluated using a two-tailed Student's *t*-test for continuous variables with a normal distribution, and the Mann–Whitney *U* test for continuous variables with a non-normal distribution or a sample size less than or equal to five. In addition, maternal age and pre-gestational BMI were included in the analysis of qRT-PCR results for UCA1 expression. *P* < 0.05 was considered statistically significant, and in vitro experiments were repeated at least three times.

## Data Availability

The data that support the findings of this study are available from the corresponding author upon reasonable request.

## Abbreviations

HIF1*α*: Hypoxia-inducible factor 1 subunit alpha
lncRNA: Long noncoding RNA
UCA1: Urothelial cancer associated 1
VEGF: Vascular endothelial growth factor
HK2: Hexokinase 2
PKM: Pyruvate kinase M
ROS: Reactive oxygen species
PFN1: Profilin-1
ROCK: Rho-kinase
vWF: von Willebrand factor
TM: Thrombomodulin
ET: Endothelin
USP14: Ubiquitin-specific peptidase 14
UBA1: Ubiquitin-like modifier activating enzyme 1
UBA52: Ubiquitin-like modifier activating enzyme 52
CHX: Cycloheximide.

## Supplementary Materials

Supplementary Materials Table S1. The clinical characteristics between women in early gestation and women in full-term pregnancy with normal blood pressure. Table S2. The clinical characteristics of maternal serum samples derived from preeclamptic women and normotensive pregnant women. Table S3. Primer sequences used in experiments. Table S4. Antibodies used for WB and RIP. Figure S1. Species conservation of hypoxia associated lncRNAs. Figure S2. mRNA level of HIF1*α* in trophoblast cells incubated with CoCl_2._ Figure S3. The efficacy of overexpression and knockdown of UCA1. Figure S4. The DEGs after UCA1 overexpression in HUVECs.
