# Supplementary material for: Trophoblast Exosomal UCA1 Induces Endothelial Injury through the PFN1-RhoA/ROCK Pathway in Preeclampsia: A Human-Specific Adaptive Pathogenic Mechanism
Source: Oxid Med Cell Longev. 2022 Sep 15;2022:2198923. doi: 10.1155/2022/2198923 (PMC9499815; doi:10.1155/2022/2198923)
Supplement: Supplementary Materials — Table S1. The clinical characteristics between women in early gestation and women in full-term pregnancy with normal blood pressure. Table S2. The clinical characteristics of maternal serum samples derived from preeclamptic women and normotensive pregnant women. Table S3. Primer sequences used in experiments. Table S4. Antibodies used for WB and RIP. Figure S1. Species conservation of hypoxia associated lncRNAs. Figure S2. mRNA level of HIF1α in trophoblast cells incubated with CoCl2. Figure S3. The efficacy of overexpression and knockdown of UCA1. Figure S4. The DEGs after UCA1 overexpression in HUVECs. [file 2198923.f1.docx]

| **Table S1. The clinical characteristics between women in early gestation and women in full-term pregnancy with normal blood pressure.** | | | | |
| --- | --- | --- | --- | --- |
| **Variables** | **Early gestation(n=20)** | | **Term (n=20)** | ***p* value** |
| Maternal age (years) | | 30.9 ± 4.3 | 31.9 ± 4.3 | 0.551 |
| Pre-gestational BMI (kg/m2) | | 23.3 ± 3.0 | 23.4 ± 3.2 | 0.608 |
| Values shown are means ± SD. BMI, body mass index.  **Table S2. The clinical characteristics of maternal serum samples derived from preeclamptic women and normotensive pregnant women.**   \| **Variables** \| **Control (n=20)** \| **Preeclampsia (n=20)** \| ***p* value** \| **Adjusted *p* value*** \| \| --- \| --- \| --- \| --- \| --- \| \| Maternal age (years) \| 31.9 ± 4.3 \| 31.2 ± 4.0 \| 0.601 \| - \| \| Gestational age at delivery (weeks) \| 38.8 ± 0.7 \| 35.6 ± 2.8 \| <0.001 \| - \| \| Pre-gestational BMI (kg/m2) \| 23.4 ± 3.2 \| 21.2 ± 4.1 \| 0.068 \| - \| \| Highest SBP (mm Hg) \| 122.0 ± 8.1 \| 167.4± 14.2 \| <0.001 \| <0.001 \| \| Highest DBP (mm Hg) \| 74.8 ± 7.7 \| 103.6 ± 9.2 \| <0.001 \| <0.001 \| \| Proteinuria (g/24h) \| NA \| 2.7 ± 2.1 \| NA \| - \| \| Birth weight (g) \| 3568.5 ± 571.5 \| 2408.5 ± 670.3 \| <0.001 \| <0.001 \|   Values shown are means ± SD. *The blood pressure and birth weight were also adjusted for maternal age, pre-gestational BMI and gestational age at delivery. BMI, body mass index; NA, not applicable; SBP, systolic blood pressure; DBP, diastolic blood pressure.  **Table S3. Primer sequences used in experiments.**   \| Symbol \| Primer \| Primer Sequence (5’-3’) \| \| --- \| --- \| --- \| \| UCA1# \| Forward primer \| TTTGCCAGCCTCAGCTTAAT \| \|  \| Reverse primer \| TTGTCCCCATTTTCCATCAT \| \| UCA1# \| Forward primer \| CTCTCCTATCTCCCTTCACTGA \| \|  \| Reverse primer \| CTTTGGGTTGAGGTTCCTGT \| \| HIF1α# \| Forward primer \| GAACGTCGAAAAGAAAAGTCTCG \| \|  \| Reverse primer \| CCTTATCAAGATGCGAACTCACA \| \| β-actin# \| Forward primer \| AGAGCTACGAGCTGCCTGAC \| \|  \| Reverse primer \| AGCACTGTGTTGGCGTACAG \| \| β-actin# \| Forward primer \| CATGTACGTTGCTATCCAGGC \| \|  \| Reverse primer \| CTCCTTAATGTCACGCACGAT \| \| U1# \| Forward primer \| GACGGGAAAAGATTGAGCGG \| \|  \| Reverse primer \| GCCACGAAGAGAGTCTTGAAGG \| \| GAPDH# \| Forward primer \| AGTGGCAAAGTGGAGATT \| \|  \| Reverse primer \| GTGGAGTCATACTGGAACA \| \| U6# \| Forward primer \| CTCGCTTCGGCAGCACA \| \|  \| Reverse primer \| AACGCTTCACGAATTTGCGT \| \| PFN1# \| Forward primer \| CTGTCAGGACGCGGCCATCG \| \|  \| Reverse primer \| CAGCTGGCGTGATGTTGACGA \| \| UCA1 Sense-1$ \| Forward primer \| taatacgactcactataggg TGACATTCTTCTGGACAATGAGTCCCATCA \| \|  \| Reverse primer \| GGCTGGCAAAGAGTGAAATGTCCCAAGCCC \| \| UCA1Antisense-1$ \| Forward primer \| taatacgactcactataggg GGCTGGCAAAGAGTGAAATGTCCCAAGCCC \| \|  \| Reverse primer \| TGACATTCTTCTGGACAATGAGTCCCATCA \| \| UCA1 Sense-2$ \| Forward primer \| taatacgactcactataggg GTATGTTGTTTGTTGTTAGAGGGCTTGGGA \| \|  \| Reverse primer \| CTGACTCTTTTAGGAAGATTTCTTTTCTGTCACCT \| \| UCA1Antisense-2$ \| Forward primer \| taatacgactcactataggg TGACTCTTTTAGGAAGATTTCTTTTCTGTCACCT \| \|  \| Reverse primer \| GTATGTTGTTTGTTGTTAGAGGGCTTGGGA \|   # Used only for qRT-PCR; $ used for RNA pulldown assay.  **Table S4. Antibodies used for WB and RIP.**   \| **Name** \| **Antibody number** \| **Concentration** \| \| --- \| --- \| --- \| \| **β-actin** \| ProteinTech Group, Inc. \| 1:2000 \| \| **HIF1α** \| Cell Signaling Technology  36169 \| 1:1000 \| \| **HK2** \| Cell Signaling Technology  2867 \| 1:1000 \| \| **PKM** \| Cell Signaling Technology  4053 \| 1:1000 \| \| **GLUT3** \| ProteinTech Group, Inc.  20403-1-AP \| 1:1000 \| \| **LDHA** \| Cell Signaling Technology  3582 \| 1:1000 \| \| **TSG101** \| Abcam ab125011 \| 1:1000 \| \| **HSP90** \| Cell Signaling Technology  4877 \| 1:1000 \| \| **HSP70** \| Abcam ab181606 \| 1:1000 \| \| **Thrombomodulin** \| Abcam ab109189 \| 1:5000 \| \| **Von Willebrand Factor** \| Abcam ab154193 \| 1:5000 \| \| **p-RhoA (S188)** \| Abcam ab41435 \| 1:1000 \| \| **RhoA** \| Abcam ab187027 \| 1:5000 \| \| **ROCK** \| Abcam ab45171 \| 1:5000 \| \| **Profilin1** \| Abcam ab124904 \| 1:10000 \| \| **USP14** \| ProteinTech 14517-1-AP \| 1:1000 \| \| **Ubiquition** \| ProteinTech 10201-2-AP \| 1:1000 \| \| **Secondary antibody- Goat anti mouse** \| ProteinTech Group, Inc. \| 1:5000 for β-actin;  1:2000 for others; \| \| **Secondary antibody- Goat anti rabbit** \| ProteinTech Group, Inc. \| 1:2000 \| | | | | |


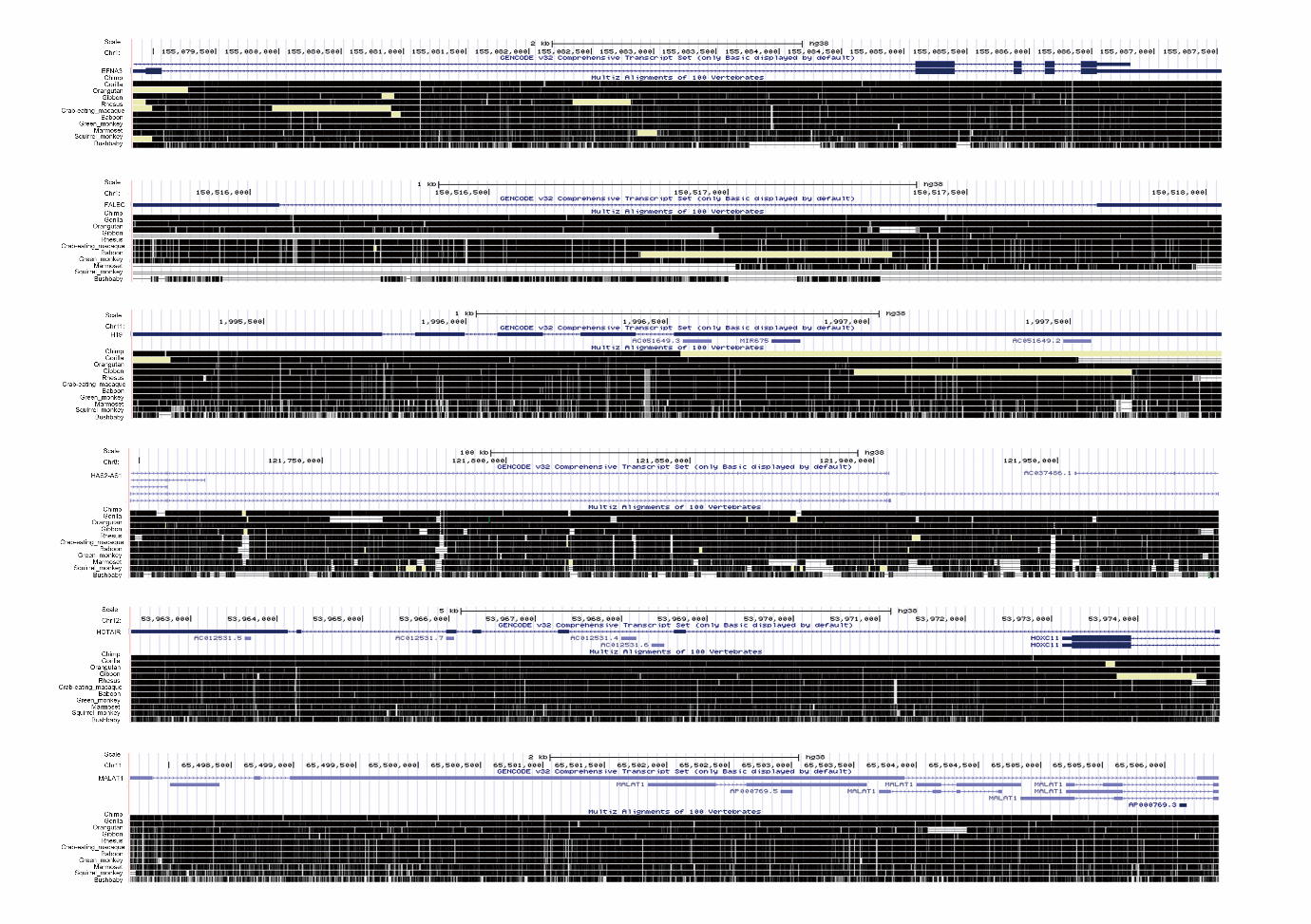


**Fig. S1 Species conservation of hypoxia associated lncRNAs.**

Evolutionary analysis of EFNA3, H19, MALAT1, HOTAIR, FALEC and HAS2-AS1 sequences from UCSC database, showing that the conservation of these lncRNAs was poorly matched to the species specificity of preeclampsia.


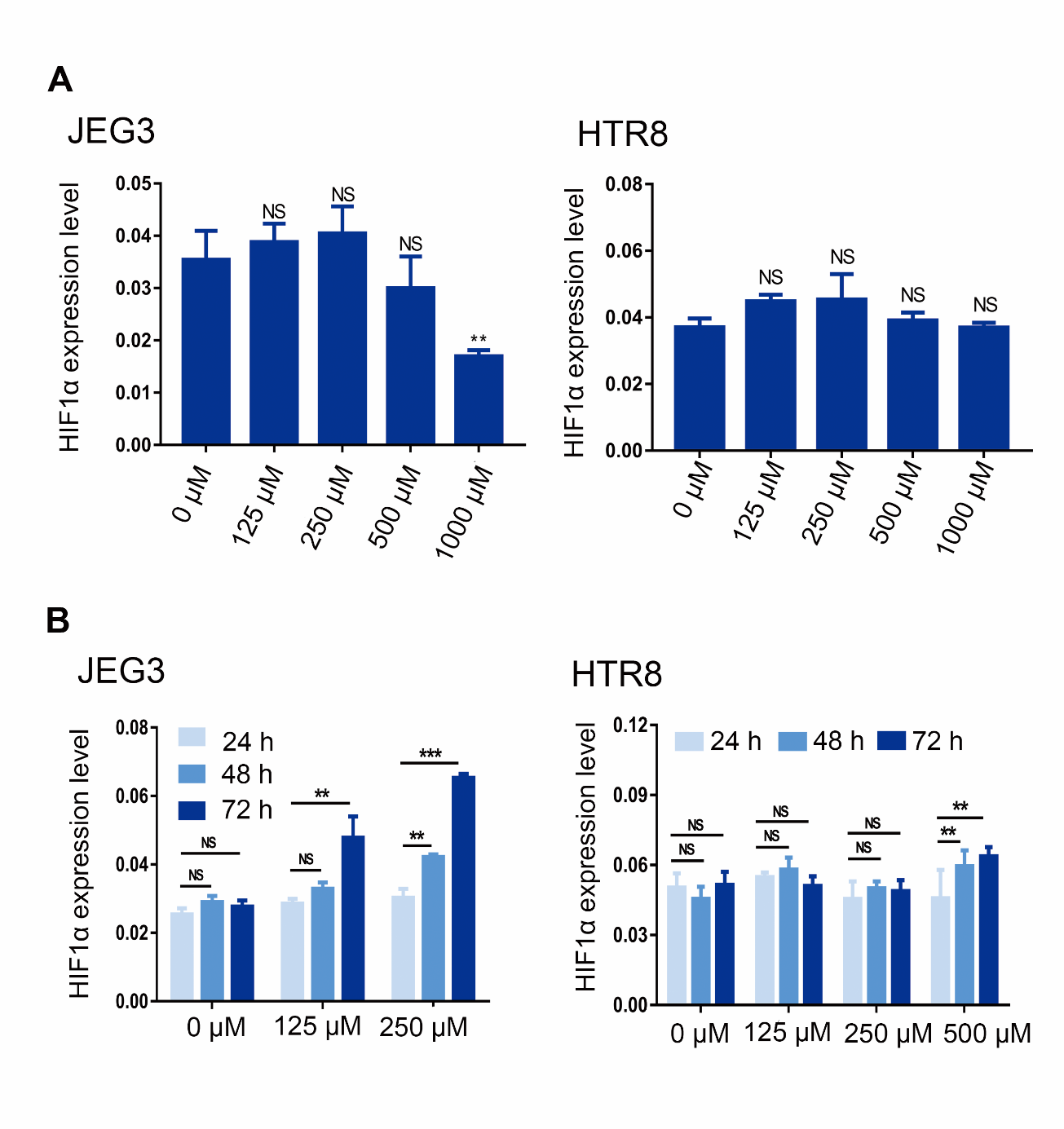


**Fig. S2 mRNA level of HIF1α in trophoblast cells incubated with CoCl_2._**

mRNA expression level of HIF1α in HTR-8/Svneo and JEG-3 cells incubated with different concentrations (A) or time periods (B) of CoCl_2._ Data were calculated with 2^−ΔΔCt^. **P<0.01. ***P<0.001. NS, no significance.


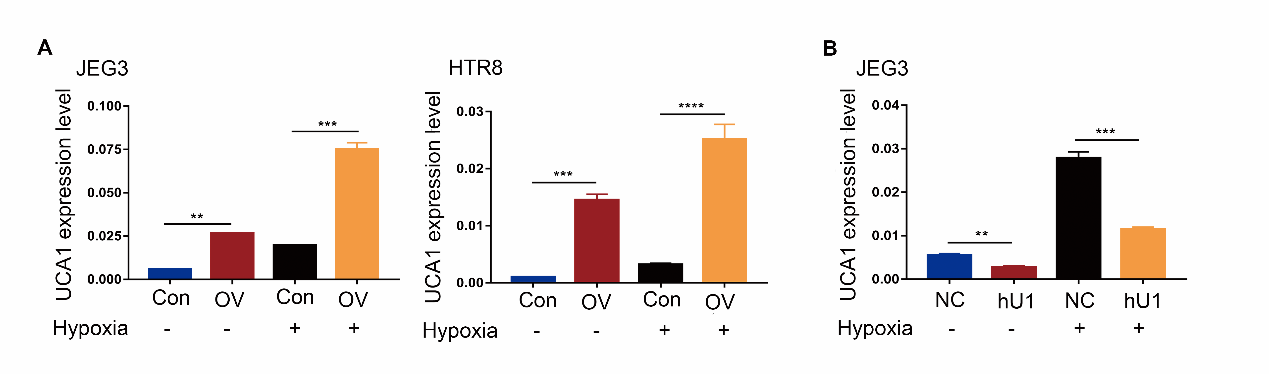


**Fig. S3 The efficacy of overexpression and knockdown of UCA1.**

(A) Overexpression efficacy of UCA1 in HTR-8/Svneo (right) and JEG-3 cells (left). (B) Knockdown efficacy of UCA1 in JEG3 cells.


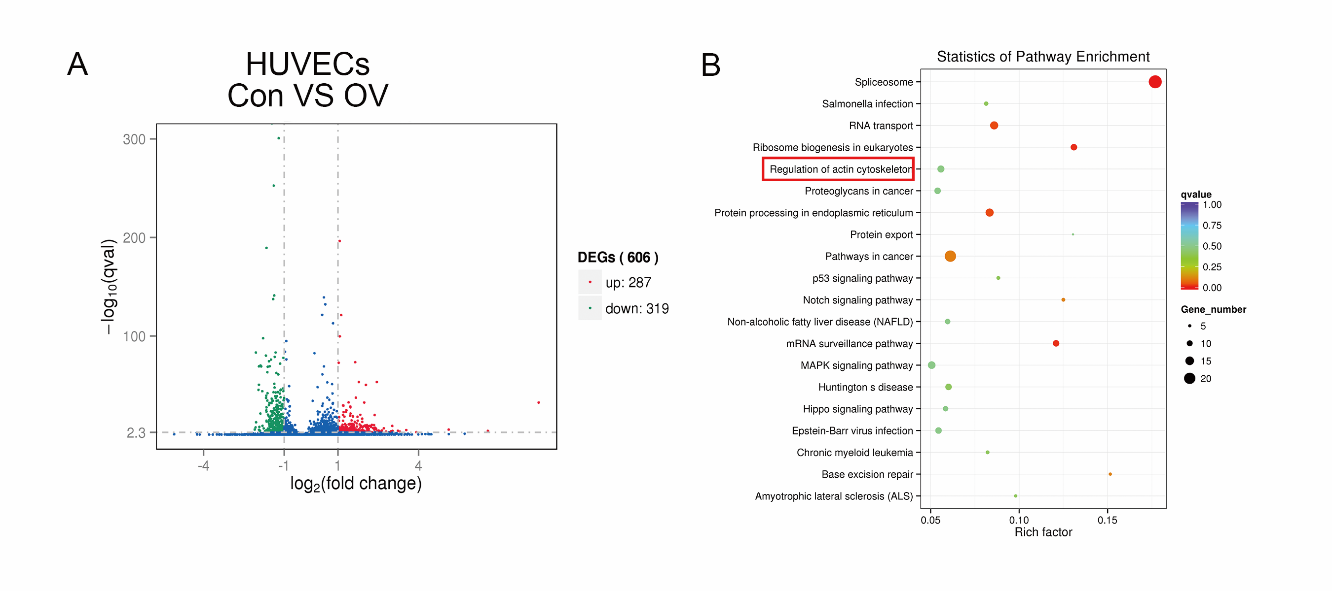


**Fig. S4 The DEGs after UCA1 overexpression in HUVECs.**

(A) Volcano plot of differentially expressed genes after UCA1 overexpression. The red dots indicated the upregulated genes, and the green dots indicated the downregulated genes. The threshold value was set as |log2(Foldchange)| > 1 and q value<0.005. (B) Scatterplot of KEGG pathways. The ordinate represented the name of pathway, and the abscissa represented the rich factor.
